# Volumetric Modification of Transparent Materials with Two-Color Laser Irradiation: Insight from Numerical Modeling

**DOI:** 10.3390/ma17081763

**Published:** 2024-04-11

**Authors:** Vladimir P. Zhukov, Nadezhda M. Bulgakova

**Affiliations:** 1 HiLASE Centre, Institute of Physics ASCR, 25241 Dolni Brezany, Czech Republic; zukov@ict.nsc.ru; 2 Federal Research Center for Information and Computational Technologies, Novosibirsk 630090, Russia; 3 Novosibirsk State Technical University, Novosibirsk 630073, Russia

**Keywords:** ultrashort laser pulses, material modification, bi-color irradiation, laser energy coupling, numerical simulations, Maxwell’s equations

## Abstract

Traditionally, single-color laser beams are used for material processing and modifications of optical, mechanical, conductive, and thermal properties of different materials. So far, there are a limited number of studies about the dual-wavelength laser irradiation of materials, which, however, indicate a strong enhancement in laser energy coupling to solid targets. Here, a theoretical study is reported that aimed at exploring the volumetric excitation of fused silica with dual-wavelength (800 nm and 400 nm) ultrashort laser pulses focused on the material’s bulk. Numerical simulations are based on Maxwell’s equations, accounting for the generation of conduction electrons, their hydrodynamic motion in the laser field, and trapping into an excitonic state. It is shown that, by properly choosing the energies of the two laser harmonics successively coupling with the material, it is possible to strongly enhance the laser energy absorption as compared to the pulses of a single wavelength with the same total energy. Laser energy absorption strongly depends on the sequence of applied wavelengths, so that the shorter wavelength pre-irradiation can yield a dramatic effect on laser excitation by the following longer-wavelength pulse. The predictions of this study can open a new route for enhancing and controlling the highly localized absorption of laser energy inside transparent materials for optoelectronic and photonic applications.

## 1. Introduction

Nowadays, femtosecond lasers are becoming a versatile tool not only for the scientific research of ultrafast phenomena but also for many applications in industry and biomedicine [1,2,3,4], among which the direct writing of 3D devices based on transparent materials has great potential for optoelectronics, photonics, and sensing technologies [3,5,6,7]. The advantage of ultrashort laser pulses over longer ones is the possibility of localizing laser energy absorption while minimizing the heat-affected zone [1]. For optical glasses having band gaps of several electron volts, the absorbed energy localization is amplified by the nonlinear photoionization effect [8]. However, to ensure 3D laser writing techniques reach the level of routine industrial instruments, a wide scope of research is still required. It is necessary to learn how laser energy can be coupled in transparent dielectrics most efficiently, how to precisely predict and control the material damage/restructuring at a desired level and at the smallest volume, and, for some applications, how to reach the highest possible stresses [9].

Traditionally, single-color laser beams are used for material processing; modifications of the structural, optical, mechanical, conductive, and thermal properties of different materials (both on the surface and inside the bulk); and for designing new nanostructured material systems. However, several studies have reported the advantages of dual-wavelength laser irradiation for surface modification as compared to single-color beams. Thus, it was shown that a small fraction of the second harmonics added to the fundamental one can strongly improve the quality of laser-induced periodic surface structures (LIPSSs) on semiconductor surfaces [10]. The efficiency of metal nanoparticle production was demonstrated to be enhanced using two-color, two-laser irradiation as compared to single-wavelength light [11]. The application of dual-wavelength irradiation for pulsed laser deposition can improve the quality of the deposited thin films [12]. Zoppel et al. [13] achieved a 70% enhancement in the laser ablation yield of silicon by adding a small amount of the second harmonics to the fundamental ablating one. The microfabrication of optical structures in hard materials can be substantially improved using multiwavelength processing [14]. Enhanced efficiency of drilling using dual laser frequencies has been reported for metals [15] and soft materials [16]. Better control over femtosecond laser modification inside BK7 glass was demonstrated when adding CO_2_ laser radiation [17]. Significantly higher density of the conduction-band electrons was reported for a case of two-color femtosecond laser excitation inside fused silica in comparison with single-color pulsed irradiation [18]. However, the question of the benefits of dual-wavelength irradiation for laser material processing remains insufficiently studied. It was shown that some bi-color irradiation regimes, although successful at first glance, are energetically impractical [19]. Gaudfrin et al. [20] reported that the laser ablation of fused silica by ultrashort laser pulses at 1030 and 515 nm wavelength does not show a benefit over single-pulse 515 nm irradiation. Recently, a detailed experimental and theoretical study of laser-induced damage and ablation of silicon was performed using pairs of femtosecond pulses of different wavelengths, 1030 and 515 nm, to address the physical mechanisms of bi-color ablation of silicon and explore possibilities to increase the ablation efficiency [21]. It was shown that the order in which the pulses couple with the material is essential in bi-color ablation, with higher material removal rates when a shorter-wavelength pulse arrives first at the surface. There have also been several theoretical attempts to calculate the bi-color laser excitation of bandgap materials using ab initio approaches such as the optical Bloch equation [22] and the time-dependent density functional theory (TDDFT) [23]. Such calculations enable in-depth insights into ultrafast processes at the nanoscale, pointing out the potential advantages of bi-color irradiation regimes due to enhanced light absorption as compared to the single wavelength. However, the introduction of ab initio simulations into the real spatiotemporal modeling of the laser light propagation inside band-gap materials is not feasible because of limited computer resources, both in terms of performance and memory.

Our preliminary study indicated that dual-wavelength irradiation can be seen as one of the solutions to make modifications more localized and controllable. This work aims to theoretically investigate the possible effects of bi-color laser pulses on the volumetric modification of fused silica in the regimes of 3D direct laser writing. The propagation of focused laser beams inside transparent solids is usually described by models based on either the nonlinear Schrödinger equation [24,25,26] or Maxwell’s equations [27,28,29,30]. Both approaches have been proven to adequately describe the dynamics of the complex phenomenon of laser light absorption in ionizable media and to predict the strength of the laser energy coupling and its geometry in a wide range of irradiation conditions. It is natural that the previously developed model, which is based on Maxwell’s equations [28,30], was used in the present study. The model takes into account the linear and nonlinear features of the focused laser beam propagation in transparent dielectrics with photoionization of the material, collisional multiplication of the conduction band electrons, and electron trapping into the excitonic states. Numerical simulations were performed for two temporally separated femtosecond laser pulses of a Ti:sapphire laser at the fundamental wavelength (*λ*_0_ = 800 nm) and second harmonics (*λ*_0_ = 400 nm). The regimes were revealed in which a dramatic enhancement in laser energy coupling into a highly localized zone in material bulk occurs. However, with increasing pulse energies of either one or both harmonics, the efficiency of laser energy coupling drops, and the laser-excited region increases. The findings of this work can be used for optimization of the direct laser writing of photonic structures inside optical glasses.

## 2. Mathematical Model

Numerical simulations of fused silica irradiation for with dual-wavelength femtosecond laser pulses were performed based on nonlinear Maxwell’s equations supplemented by the rate equations describing the excitation of electrons from the valence to the conduction band and their trapping and by the hydrodynamic-like equations describing the oscillations of the conduction electrons in the field of the laser wave. The complete modeling equations can be found in [28,30]. Here, only the key equations are presented, which read as follows:

(1) $$\frac{1}{c}\frac{\partial D}{\partial t}-i\frac{\omega }{c}D=\frac{4\pi }{c}e\rho v+rot\;B-\frac{8\pi }{c{\left|E\right|}^{2}}\alpha \hbar \omega W_{PI}E,$$

(2) $$D=n^{2}\left(1+\frac{cn_{2}{\left|E\right|}^{2}}{4\pi }\right)E,$$

(3) $$\frac{1}{c}\frac{\partial B}{\partial t}-i\frac{\omega }{c}B=-rot\;E$$

(4) $$\frac{\partial \rho }{\partial t}=W_{PI}+W_{\sigma }-\frac{\rho }{\tau_{tr}},$$

(5) $$i\omega v=\left(e/m_{e}\right)E+\frac{v}{\tau_{c}}$$

Here, ***E***, ***B***, and ***D*** correspond, respectively, to the electric and magnetic fields of the laser wave and the electric displacement field; *ω* is the laser pulse frequency at 800 nm or 400 nm; *c* is the speed of light; *ρ* and v denote the density and the velocity of the electrons excited to the conduction band; *e* is the elementary charge. $W_{PI}$ and $W_{\sigma }$ are the multiphoton and collisional ionization rates. The coefficients in the $W_{PI}$ rates for 800 nm and 400 nm were derived from estimations based on Keldysh photoionization theory [31] for relatively low intensities, which are, respectively, equal to 3.1 × 10^34^ cm^−3^s^−1^ and 6.3 × 10^36^ cm^−3^s^−1^. It should be underlined that the two-color simulations were performed here only for laser pulses separated in time so that the photoionization by the simultaneous action of two wavelengths would not apply. Such a nonoverlapping pulsed regime appears to be more advantageous for laser energy coupling inside bulk transparent materials as, for overlapped pulses, the local absorption efficiency can decrease due to the free-electron plasma shielding effect [32]. However, for overlapped bi-color pulses, it is necessary to introduce mixed wavelength ionization rates [22,23] which are still unknown.

The orders of multiphoton ionization of fused silica, α, are equal to 6 and 3, respectively, for 800 nm and 400 nm irradiation; $m_{e}$ is the electron mass; $\tau_{c}$ and $\tau_{tr}$ are the electron collision time and the electron trapping time (1.28 fs and 150 fs, respectively). In most simulations, re-excitation of the self-trapped electrons (STEs) by laser radiation was not considered in this study intentionally to explore the role of light absorption by free electrons in the inverse bremsstrahlung process, which can lead to collisional multiplication of the electrons. n and $n_{2}$ are the linear and nonlinear refractive indices. For 800 nm radiation, n = 1.45 and $n_{2}$ = 2.48 × 10^16^ cm^2^W^−1^, while for the 400 nm wavelength, they are 1.47 and 3.5 × 10^16^ cm^2^W^−1^, respectively.

All simulations reported below were performed for the following irradiation conditions. Both pulses were focused to a depth f of 120 µm below the sample surface. The Gaussian laser pulses entering the sample had linear polarization (along the x-axis), and their focusing is described by the following expression:

(6) $$E_{x}=E_{0}exp\left(-i\omega t-t^{2}/\tau_{L}^{2}-r^{2}/w^{2}-{i\pi nr}^{2}/(\lambda_{0}f)\right)$$

The width of the pulse upon entering the sample was calculated for both wavelengths for the corresponding numerical apertures, NA = 0.125 for 800 nm and NA = 0.25 for 400 nm, with beam waists of 2 μm and 0.509 μm, respectively. Pulse durations $\tau_{L}$ were 180 fs (800 nm) and 140 fs (400 nm). Further details on the modeling equations and used parameters can be found in [30]. The main parameter of interest in this study was the absorbed laser energy density $E_{ab}$, as this value determines the material’s modification level. To map $E_{ab}$ within the laser-affected region inside fused silica, the following integration was performed during the simulations:

(7) $$E_{ab}=\int_{t_{0}}^{t_{fin}}\left(\frac{jE^{*}+j^{*}E}{4}+\alpha \hbar \omega W_{PI}\right)dt$$

This integral was calculated for each cell of the numerical grid from the start of the simulation ($t_{0}$) when the first laser pulse entered the sample till the time moment $t_{fin}$ when the second laser beam left the focal zone so that further light absorption became negligible.

## 3. Results and Discussion

The simulations were performed for two ultrashort laser pulses at fundamental and second harmonics of a Ti:sapphire laser (central wavelengths of 800 nm and 400 nm). The pulses were separated in time so that they did not overlap. Below, most of the results for double pulses are given for the separation time $t_{s}$ of 800 fs. The impact of the separation time on laser energy absorption is discussed below.

**Figure 1:**
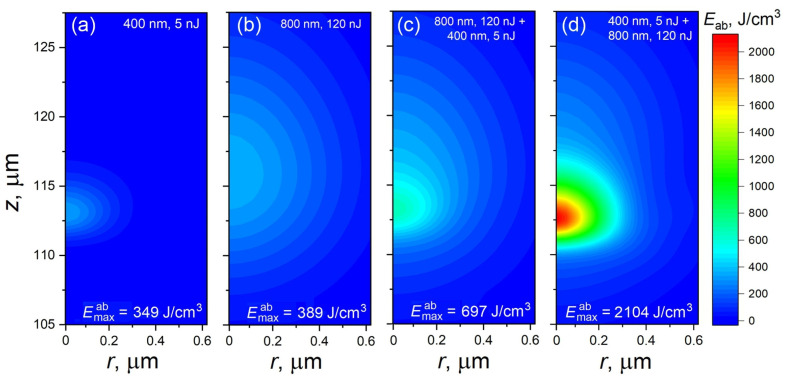
Demonstration of the efficiency of two-color laser irradiation of bulk silica glass at small pulse energies. The absorbed energy density maps for the action of a single 5 nJ laser pulse at 400 nm wavelength (**a**), a single 120 nJ laser pulse at 800 nm wavelength (**b**), and dual-wavelength irradiation when 800 nm (**c**) and 400 nm pulses (**d**) come to the focal zone first. The laser pulse propagates from the bottom. The geometrical focus of both pules was located at a depth of 120 μm from the sample surface. The time separation for double pulses was 800 fs.

Figure 1 presents the spatial distributions of $E_{ab}$ for the 5 nJ laser pulse at the second harmonic (SH) (a), the 120 nJ pulse at the 800 nm wavelength (b), and the sequence of these two pulses with the fundamental pulse coupling first (c) and with the SH coupling first (d). Single 5 nJ and 120 nJ pulses, (a) and (b), result in rather gentle heating, with absorption maxima of ~113 μm and ~116.5 μm, respectively, from the sample surface. Interestingly, the maximum values of the absorbed energy density $E_{\mathrm{ab}}^{\max}$ are close for these two regimes. According to thermodynamic considerations [33], the maximum rise in the sample temperature in the laser-affected zones does not exceed ~220–240 K, while the SH excitation is much more strongly localized toward nanoscale dimensions as compared to the 800 nm excitation. For the double-pulse irradiation regime, when the 800 nm pulse interacts with the sample first (c), the $E_{max}^{ab}$ value is smaller than the additive $E_{max}^{ab}$ from two single pulses at 400 nm and 800 nm with the evaluated temperature rise of ~430 K (note that the glass transition point of fused silica above which one can expect to observe signs of modification is ~1480 K). This effect of nonadditivity can be explained by the delocalization of the SH pulse by the free-electron plasma created by the 800 nm pulse, which can scatter the second pulse photons to the periphery. Although the effect is slight, an increase in the heat-affected zone is clearly visible in Figure 1c.

The situation overturned dramatically when the 400 nm laser pulse comes first (Figure 1d). In such a regime, the $E_{max}^{ab}$ value is strongly amplified, almost three times as compared to the additive value from two separate pulses. The maximum temperature achieved in the laser-affected zone can be evaluated as ~1615 K, which is above the glass transition point, and, hence, the modification threshold is exceeded. Thus, a violet pre-pulse with only 5 nJ energy strongly stimulates laser energy coupling from a near-infrared laser pulse. This is explained by the fact that such a pre-pulse efficiently excites electrons from the valence to the conduction band. The following IR pulse is efficiently absorbed by the conduction-band electrons, which were generated by the pre-pulse, via the inverse bremsstrahlung process. The rate of absorption is proportional to the square of the wavelength [34], so the pulses at the fundamental harmonics are much better absorbed as compared to the SH pulses (compare Figure 1c,d). In Figure 1d, one can also observe some increase in the laser-affected region and a distortion of the absorbed energy levels in the periphery, which can be attributed to IR light scattering by the laser-excited free-electron plasma. Also, the maximum density of the absorbed laser energy is the same as that of the pre-pulse (Figure 1a), as the absorption of the IR pulse is mostly governed by the bremsstrahlung mechanism due to the population of free electrons generated by pre-pulse.

It should be mentioned that the shift in the region of maximum absorption from the geometrical focus (120 μm from the sample surface) toward the laser (Figure 1) is not connected with the self-focusing effect. Although the Kerr effect is naturally taken into account in our model (see Equation (2)), the power of laser pulses in Figure 1 is well below the critical power for self-focusing $P_{cr}=3.72\lambda_{0}^{2}/\left(8\pi nn_{2}\right)$ [26,35]. Indeed, for fused silica, the $P_{cr}$ values are ~2.63 MW and 0.46 MW for 800 nm and 400 nm, respectively. The power of 5 nJ 400 nm pulses is only ~35.7 kW, and the power of 120 nJ 800 nm pulses is ~0.67 MW. The observed shift in absorption can be explained as follows: Upon the focusing of the laser beam, the efficient photoionization of a bandgap material starts as soon as the beam intensity reaches a certain value sufficient for the photoexcitation of electrons from the valence to the conduction band. Depending on the beam energy and numerical aperture, this happens at different distances before the geometrical focus. The photoionization process as well as the light absorption by generated free electrons (the inverse bremsstrahlung process) consume the energy of the laser beam, which leads to the so-called intensity clamping effect [36,37,38] when an intensity upper limit is set along the beam propagation that also limits the size of the material’s ionization region. For low-energy beams, this can lead to beam energy depletion so that the intensity becomes insufficient to efficiently ionize material closer to the geometric focus. Specifically, this happens in the cases demonstrated in Figure 1.

**Figure 2:**
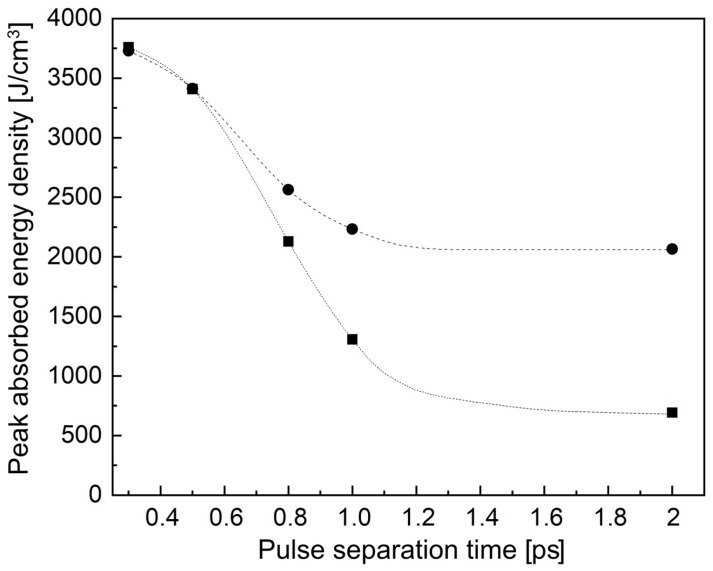
The maximum values of the absorbed laser energy density on the separation time of two pulses for the regime shown in Figure 1d (squares). Dark circles indicate the results of simulations with accounting re-excitation of self-trapped excitons by the 800 nm pulse. The lines are given to guide the eyes.

To explore the dependence of the absorbed laser energy density on the separation time between two pulses for the regime shown in Figure 1d when a very small 400 nm pre-pulse is applied followed by a 120 nJ 800 nm laser pulse, a series of simulations was performed for several $t_{s}$ of 300 fs, 500 fs, 800 fs, 1 ps, and 2 ps. To understand the contribution of STE re-excitation to laser energy absorption, the simulations were also carried out for the same pulse delays but with the consideration of the second-pulse photoionization of the STE states produced by trapping the conduction band electrons generated by the 400 nm pre-pulse. The details of the model with the re-excitation of STEs can be found in [28,30]. The peak values of the absorbed energy density $E_{\mathrm{ab}}^{\max}$ as a function of the pulse separation time are presented in Figure 2. For relatively short delays up to ~600 fs, the $E_{\mathrm{ab}}^{\max}$ values are very close for the simulation cases with and without considering STE re-excitation. This shows that the light absorption from the second laser pulse is mostly governed by the inverse bremsstrahlung process. Furthermore, the time of 600 fs agrees well with the measured characteristic decay time of the shielding effect [32]. This effect is related to an efficient absorption and scattering of the second pulse by the free electron plasma produced by the first pulse in double-pulse regimes.

With increasing separation time, the contribution of STE re-excitation becomes visible. For a $t_{s}$ = 800 fs separation time, the contribution of the energy absorption in the STE re-excitation process is ~17%, which rapidly increases with further increasing delay time and reaches ~41% at $t_{s}$ = 1 ps. Nevertheless, for all these $t_{s}$ values, the sequence of the 5 nJ 400 nm pre-pulse followed by the 120 nJ 800 nm pulse yields much higher $E_{\mathrm{ab}}^{\max}$ values than for the inverted sequence of the pulses (see Figure 1c). Interestingly, at $t_{s}$ = 2 ps, the $E_{\mathrm{ab}}^{\max}$ value calculated without the STE re-excitation effect is almost three times smaller than that obtained with STE-re-excitation (Figure 2), indicating that the material excitation process is mostly governed by photoionization, and the free-electron plasma effects are not pronounced. However, according to the simulations, bi-color excitation with a small UV/violet pre-pulse followed by an IR pulse is more favorable for fused silica excitation in all regimes considered above. Such a combination of pulses allows one, with only one double pulse, to heat material locally above the glass transition point [33] and, hence, to induce a modification of the glass matrix. For the inverted sequence of the pulses as well as for single laser pulses of the considered energies, the maximum reached temperature remains well below the glass transition point.

It should be noted that, by tuning the inter-pulse separation time, it is possible to control the $E_{\mathrm{ab}}^{\max}$ value and hence the level of modification, possibly in terms of glass matrix restructuring and the size of the modified region, which calls for further studies.

**Figure 3:**
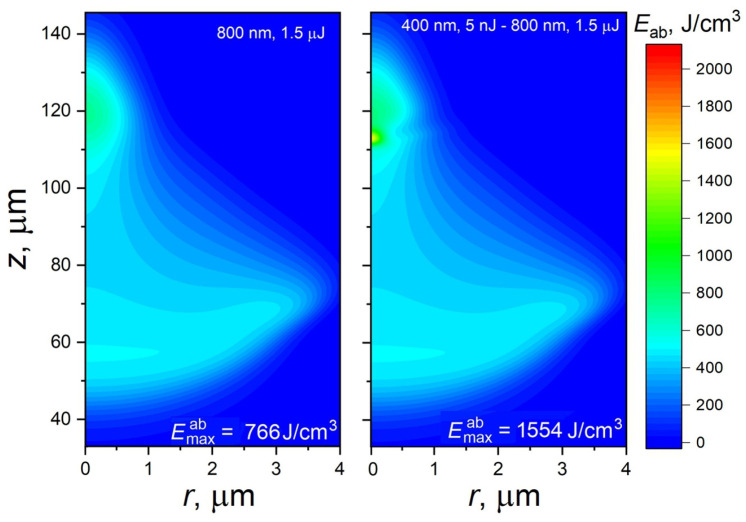
Distributions of the absorbed laser energy density for the cases of irradiation of fused silica with a single 1.5 μJ laser pulse at 800 nm wavelength (**left**) and the same pulse with a 5 nJ 400 nm pre-pulse (**right**). The other irradiation conditions are the same as those in Figure 1. Note that the bar of the absorbed laser energy is the same as that in Figure 1 for direct comparison.

It was found that, with increasing IR pulse energy, the efficiency of laser energy coupling into a localized region does not increase, while, at relatively high pulse energies, it even drops. This is demonstrated in Figure 3, where the absorbed energy maps are presented for a single IR laser pulse with an energy of 1.5 μJ (left) and for the same pulse with a 5 nJ SH pre-pulse (right). The higher the laser pulse energy, the more its absorption is delocalized, as material ionization starts earlier, before reaching the geometrical focus [30]. Thus, for a 1.5 μJ pulse, noticeable absorption already occurs ~40 μm from the sample surface, which is 80 μm before the focus. Although the zone where the absorbed energy density is maximized is located around the geometrical focus, the major $E_{ab}$ fraction is spread over a rather large volume. Application of the 5 nJ pre-pulse (Figure 3, right) still leads to an increase in $E_{max}^{ab}$. Due to this small pre-pulse, the $E_{max}^{ab}$ value increases approx. two times, from 766 J/cm^2^ to 1554 J/cm^2^, which is considerably higher than for the additive effect. However, the laser energy of the IR pulse is strongly delocalized and, according to the thermodynamic estimation [33], the maximum temperature at the $E_{max}^{ab}$ zone is ~1270 K, i.e., below the glass transition temperature of fused silica. Thus, the bi-color regime can provide high-efficiency laser energy coupling into a highly localized zone inside transparent optical materials only for the lower pulse energies of the fundamental harmonics, well below 1 μJ, with a low-energy SH pre-pulse.

**Figure 4:**
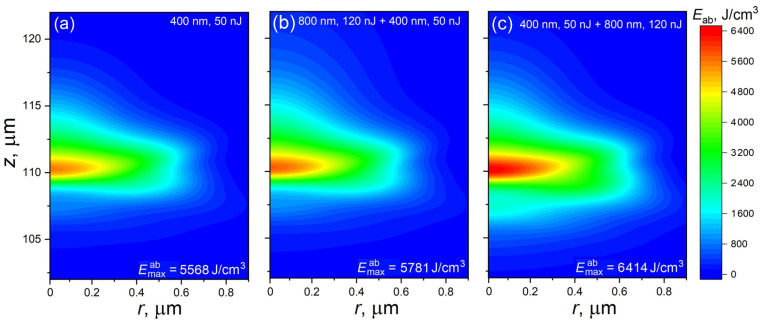
Distributions of the absorbed laser energy density for the cases of irradiation of fused silica with a single 50 nJ laser pulse at a 400 nm wavelength (**a**) and by two successive pulses with energies of 50 nJ at 400 nm and 120 nJ at 800 nm. (**b**) The IR pulse acting first and (**c**) for the reverse sequence. The other irradiation conditions are the same as those in Figure 1.

The question arises as to whether the efficiency of laser energy coupling can be enhanced by the application of a higher-energy SH pulse. The simulations show that a single 50 nJ laser pulse with a 400 nm wavelength under the irradiation conditions considered in this paper can induce strong modification with presumably the formation of voids (Figure 4a). Indeed, the locally absorbed laser energy density, being recalculated to the local temperature, can exceed 3500 K. This implies that not only material melting but also its sublimation can take place in the zone of the maximal absorption. Indeed, fused silica melts at 2006 K, its boiling temperature is 2523 K [39], and vaporization proceeds via the following reaction [40]:

2SiO_2_(*l*) → 2SiO(*g*) + O_2_(*g*) (8)

Here, *l* and *g* denote the liquid and gaseous phases. Ultrafast localized material heating with swift liquid–gas transition induces a microexplosion with the formation of a void with a densified envelope around it [41].

Now, the case of the bi-color irradiation regime is considered for the combination of 120 nJ IR and 50 nJ SH pulses (i.e., with the higher energy of the second harmonic pulse). Figure 4b,c show the $E_{ab}$ distributions for such bi-color cases. When the IR pulse acts on material first, the maximum of the absorbed laser energy density increases only slightly, below the additive value from the corresponding single pulses (compare Figure 1b and Figure 4a,b). Again, this is explained by the delocalization of the SH pulse by the free electrons created by the 800 nm pulse (see Figure 1c and the explanation of its features). When the sequence of the pulses is reversed, the effect is more significant, as the $E_{max}^{ab}$ value increases noticeably higher than for the simple additive effect, as seen from a comparison of the distributions in Figure 1b and Figure 4a,c. However, the result of the modification is expected to be qualitatively similar to that described above for the single 50 nJ pulse, which is the creation of a void structure.

## 4. Conclusions

In this work, a theoretical investigation of femtosecond laser action on fused silica in bi-color irradiation regimes was performed to explore the possible advantages of such irradiation schemes for volumetric material modification. This study is based on the numerical modeling in the frames of Maxwell’s equations supplemented with the rate equation for the generation of the conduction band electrons and the hydrodynamic-like equations describing oscillations of the electrons in the field of the laser wave. It was predicted via numerical simulations that, using only a few nJ laser energy violet laser pre-pulse, it would be possible to strongly enhance the laser energy coupling of the following IR 120 nJ laser pulse, while at the same time achieving a high localization of absorption toward the nanoscale. Although each pulse separately results only in slight glass heating, their joint action can be suitable for 3D direct writing of highly localized photonic structures inside optical glasses in a controllable and energy-saving manner. The physical mechanisms of this effect were discussed. The simulations showed that the sequence of the pulses at different wavelengths is of utmost importance and that a shorter wavelength pulse must be used as a pre-pulse to achieve enhanced energy coupling. It was also shown that increasing the pulse energies of either the IR pulse or the SH one does not lead to a valuable contribution to enhancing their joint coupling efficiency. The experimental verification of the theoretical prediction made in this study is now in progress. This work opens a new route for enhancing and controlling the highly localized absorption of laser energy inside transparent materials for optoelectronic and photonic applications, which calls for extensive studies in terms of the optimization of combined wavelengths, the ratio of energies in the bi-color pulses, and the pulse separation time.

## Data Availability

Data are contained within the article.
